# Supplementary material for: Systematic analysis on the horse-shoe-like effect in PCA plots of scRNA-seq data
Source: Bioinform Adv. 2024 Jul 29;4(1):vbae109. doi: 10.1093/bioadv/vbae109 (PMC11316618; doi:10.1093/bioadv/vbae109)
Supplement: vbae109_Supplementary_Data [file vbae109_supplementary_data.zip › Final Supplementary (PDF) V2.pdf]

Systematic analysis on the horse-shoe-like effect in PCA plots of scRNA-seq data

Najeebullah Shah[1], Qiuchen Meng[1], Zou Ziheng[1], Xuegong Zhang[1,2*]

[1]MOE Key Lab of Bioinformatics & Bioinformatics Division, BNRIST, Department of Automation, Tsinghua University, Beijing, 100084, China.
[2]Center for Synthetic and Systems Biology, Tsinghua University, Beijing, 100084, China.

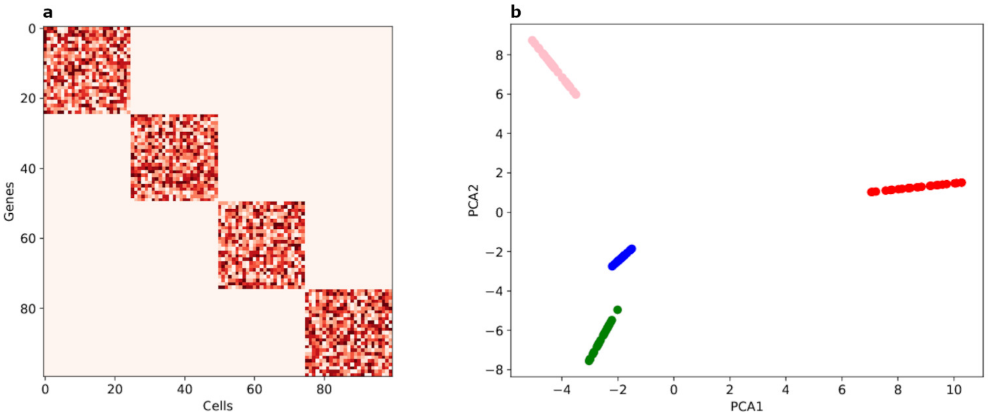

Fig. S1: (a) Simulated Cell-Gene Data Matrix with four blocks along primary diagonal, (b) 2D PC plot on Simulated Dataset with blocks along diagonal

1 Description of Simulated Band Dataset B

We created a relatively more realistic band matrix by randomly assigning count values to genes expressed along the band. Unlike the fixed value for all genes in 'Simulated

Band Dataset A', we selected count value for genes within supposed minimum and maximum values. Additionally, we structured the band so that, for the first 100 cells (columns 1 till 100 in Fig. S2a), the count value of a new gene increased from 0 in the previous cell. In simpler terms, a new gene started expressing from a non-expression state in the previous cell. Moreover, cells 101 to 200 displayed a similar pattern like in cells 1 to 100, with the addition that for cells 101 to 200, the count value of one gene became zero compared to the previous cell where the value of the same gene was non-zero. Moving on to cells 201 to 300, the only difference from the previous set of cells (101 to 200) was that genes stopped transitioning from a expressive state (count value non-zero) to a non-expressive state (count value zero). Fig. S2a illustrates the cell-gene expression/count data matrix for the described simulated dataset.

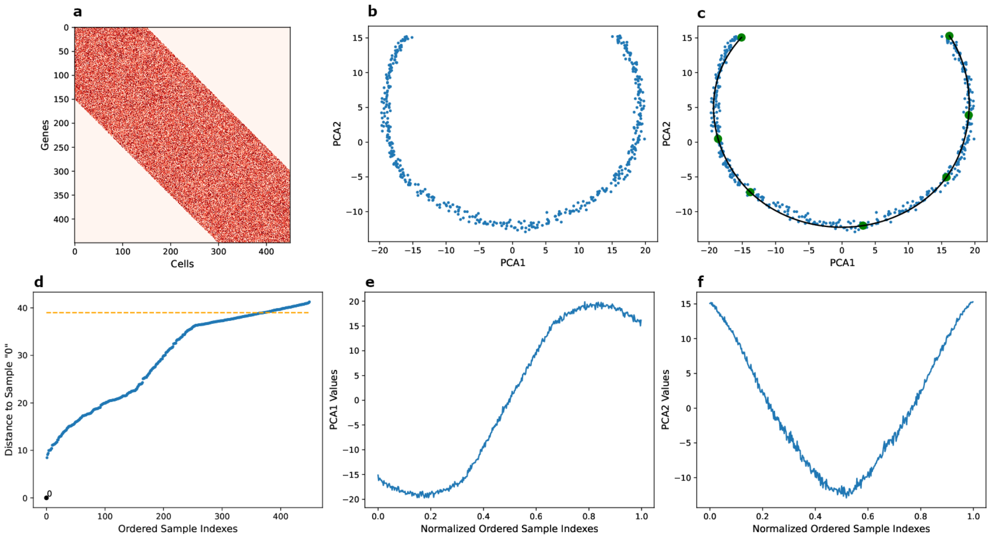

Fig. S2: (a) Ordered cell-gene data matrix, (b) 2D PC plot with horse-shoe-like effect, (c) Recovered dominant gradient superimposed on 2D PC plot, (d) 2D Distance saturation plot, (e) First eigenvector and (f) Second eigenvector for simulated band dataset B

2 Problem with the theory of Distance Saturation Property

The authors of the research work proposing that horse-shoe effect results as a consequence of distance saturation property argue that, for a particular sample, the distance to other points gradually increases along first principal component in a 2D PC plot until the euclidean distance is maximized. After reaching this point, the distance saturates, causing samples to bend around the first principal component and then follow the curve towards the second principal component, thus forming the horseshoe effect.

In our research work, we show that the distance saturation property as an explanation for inducing the horseshoe effect in PC plots, lacks sufficient clarity.

Table S1: Distance Dissimilarity for Simulated Band Dataset A

Samples	0	1	2	3	4	5	6	7	8	9	10
0	0.0	0.28	0.4	0.49	0.57	0.63	0.63	0.63	0.63	0.63	0.63
1	0.28	0.0	0.28	0.4	0.49	0.57	0.63	0.63	0.63	0.63	0.63
2	0.4	0.28	0.0	0.28	0.4	0.49	0.57	0.63	0.63	0.63	0.63
3	0.49	0.4	0.28	0.0	0.28	0.4	0.49	0.57	0.63	0.63	0.63
4	0.57	0.49	0.4	0.28	0.0	0.28	0.4	0.49	0.57	0.63	0.63
5	0.63	0.57	0.49	0.4	0.28	0.0	0.28	0.4	0.49	0.57	0.63
6	0.63	0.63	0.57	0.49	0.4	0.28	0.0	0.28	0.4	0.49	0.57
7	0.63	0.63	0.63	0.57	0.49	0.4	0.28	0.0	0.28	0.4	0.49
8	0.63	0.63	0.63	0.63	0.57	0.49	0.4	0.28	0.0	0.28	0.4
9	0.63	0.63	0.63	0.63	0.63	0.57	0.49	0.4	0.28	0.0	0.28
10	0.63	0.63	0.63	0.63	0.63	0.63	0.57	0.49	0.4	0.28	0.0

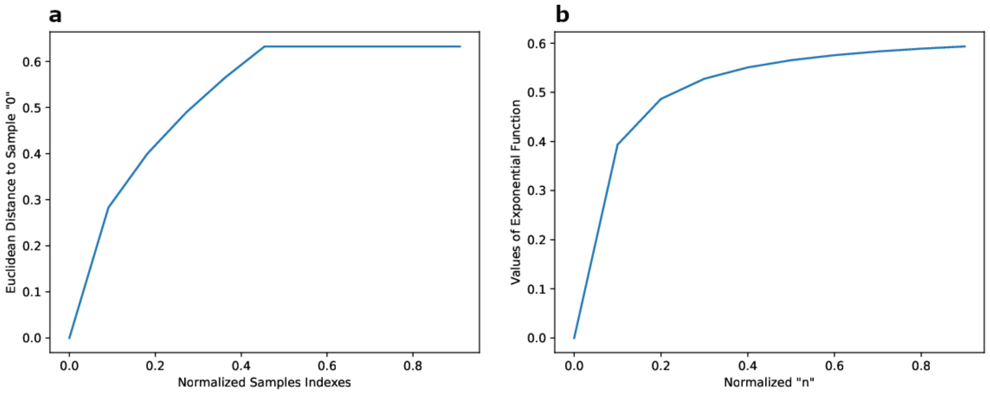

Fig. S3: (a) 2D Plot for column 1 of distance dissimilarity matrix for Simulated Band Dataset A and (b) 2D plot for column 1 for distance dissimilarity matrix with exponential terms

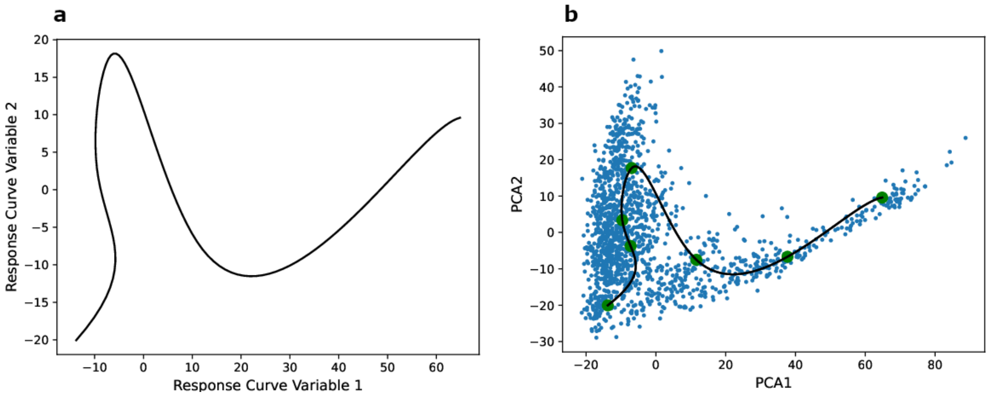

Fig. S4: (a) Estimated response curve for early human embryonic scRNA-seq developmental dataset and (b) Mapping of the estimated response curve on 2D PC plot

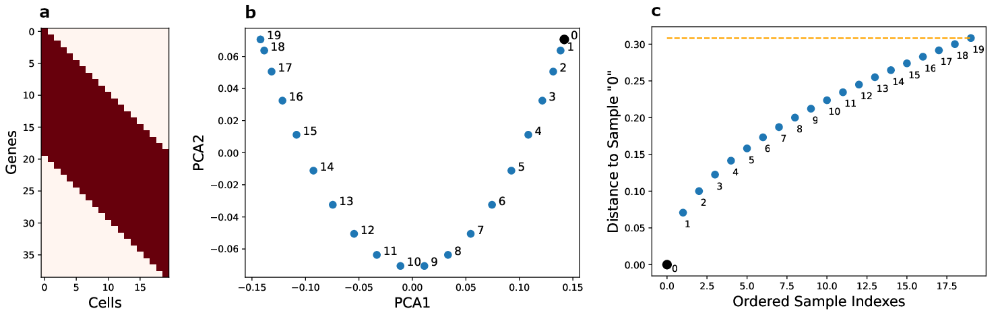

Fig. S5: (a) Simulated band data matrix with samples that does not hold distance saturation property for any sample, (b) Corresponding 2D PC plot and (c) The distance saturation plot

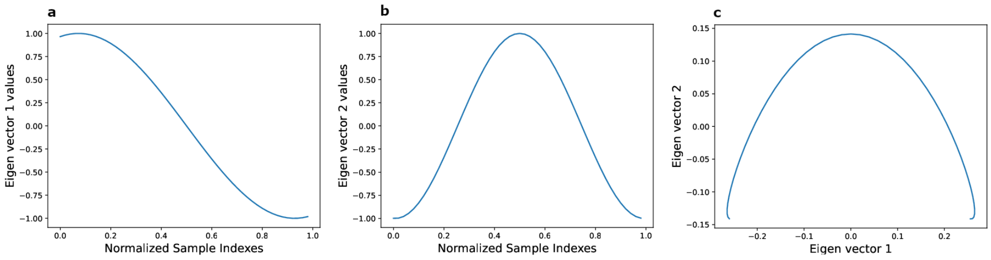

Fig. S6: (a) Estimated Eigen vector 1, (b) Estimated Eigen vector 2 derived from mathematical model and (c) The corresponding constructed 2D PCA Mapping

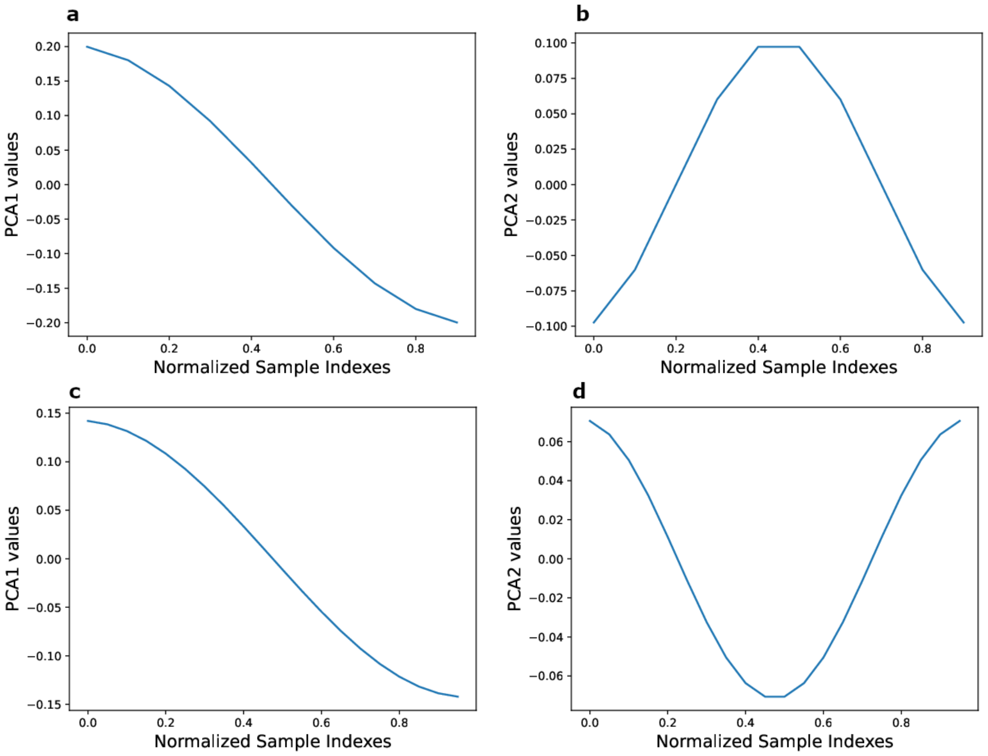

Fig. S7: (a) PC 1, (b) PC 2 for simulated dataset with 10 samples and (c) PC 1, (d) PC 2 for simulated dataset with 20 samples. Both these datasets were used to demonstrate the problem with theory of distance saturation property in relation to horse-shoe effect

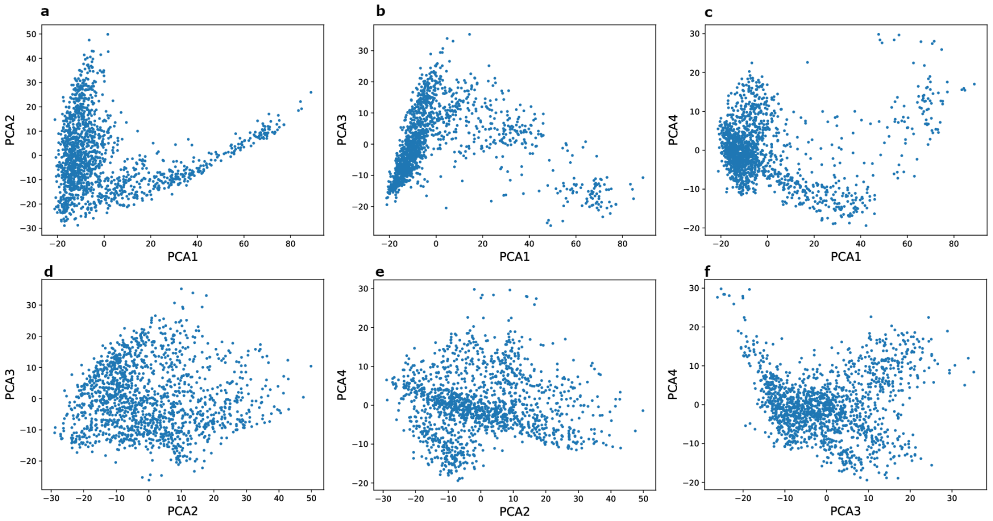

Fig. S8: 2D PCA plots of first four principal components for early human embryonic scRNA-seq datasets

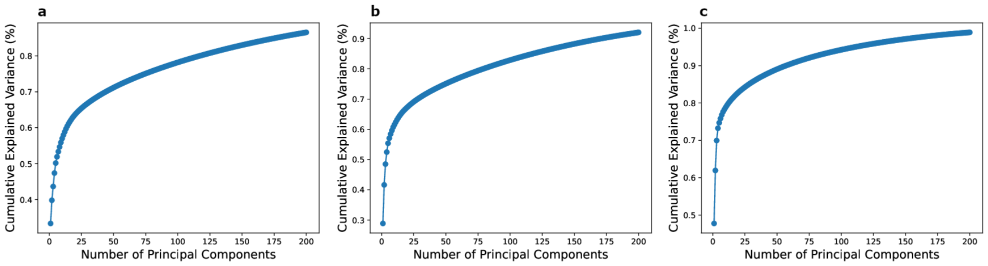

Fig. S9: Elbow plot of explained variance for principal components in (a) Liver haematopoiesis, (b) Human embryonic and (c) Mouse embryonic datasets
