# Supplementary material for: Systematic analysis on the horse-shoe-like effect in PCA plots of scRNA-seq data
Source: Bioinform Adv. 2024 Jul 29;4(1):vbae109. doi: 10.1093/bioadv/vbae109 (PMC11316618; doi:10.1093/bioadv/vbae109)
Supplement: vbae109_Supplementary_Data [file vbae109_supplementary_data.zip › Final Main Manuscript (PDF) V2.pdf]

Bioinformatics Advances, 2022, pp. 1–12

doi: DOI HERE

Advance Access Publication Date: Day Month Year

Paper

PAPER

Systematic analysis on the horse-shoe-like effect in PCA plots of scRNA-seq data

Najeebullah Shah [1], Qiuchen Meng [1], Zou Ziheng [1] and Xuegong Zhang [1,2,*]

[1]MOE Key Lab of Bioinformatics & Bioinformatics Division, BNRIST, Department of Automation, Tsinghua University, Beijing, 100084 , China and [2]School of Life Sciences and Center for Synthetic and Systems Biology, Tsinghua University, Beijing, 100084 , China

[*]Corresponding author. zhangxg@tsinghua.edu.cn

FOR PUBLISHER ONLY Received on Date Month Year; revised on Date Month Year; accepted on Date Month Year

Abstract

In single cell studies, Principal Component Analysis (PCA) is widely used to reduce the dimensionality of dataset and visualize in 2D or 3D PC plots. Scientists often focus on different clusters within PC plot, overlooking the specific phenomenon, such as horse-shoe-like effect, that may reveal hidden knowledge about underlying biological dataset. This phenomenon remains largely unexplored in single cell studies.

In this study, we investigated into the horse-shoe-like effect in PC plots using simulated and real scRNA-seq datasets. We systematically explain horse-shoe-like phenomenon from various inter-related perspectives. Initially, we establish an intuitive understanding with the help of simulated datasets. Then, we generalized the acquired knowledge on real biological scRNA-seq data. Experimental results provide logical explanations and understanding for the appearance of horse-shoe-like effect in PC plots. Furthermore, we identify a potential problem with a well-known theory of 'distance saturation property' attributed to induce horse-shoe phenomenon. Finally, we analyze a mathematical model for horse-shoe effect that suggests trigonometric solutions to estimated eigenvectors. We observe significant resemblance after comparing the results of mathematical model with simulated and real scRNA-seq datasets.

Key words: horse-shoe effect, scRNA-seq, principal component analysis, distance saturation property, ordered band tables, gradient

Introduction

Within the domain of single cell studies, single cell RNA sequencing (scRNA-seq) is used to study various cell types, understand the complex mechanism in cell-lineage developmental trajectory, among others. Technological advancements in scRNA-seq have resulted in a significant expansion of data dimensions, providing ample opportunities

© The Author 2022. Published by Oxford University Press. All rights reserved. For permissions, please e-mail: journals.permissions@oup.com

for gaining deeper insights. However, researchers and biologists use dimensionality reduction methods such as PCA , t-Distributed Stochastic Neighbor Embedding (t-SNE) [Van der Maaten and Hinton 2008], and Uniform Manifold Approximation and Projection (UMAP) [McInnes et al. 2018] to visualize the data in the reduced space. Among these techniques, PCA is preferred in terms of linearity, interpretability, reproducibility, and computational efficiency. Most importantly the ability of PCA to preserve global structures compared to t-SNE and UMAP, makes it a better option when studying the structural aspect of datasets [Huang et al. 2022].

It is important to recognize that beyond mere visualization of different clusters, the shapes delineated by PCA can yield profound insights into the underlying dataset. One of the most frequently observed shapes delineated by PCA on datasets with specific properties is the horse-shoe effect. The horse-shoe effect resembles a U-shaped curve where the second axis appears curved and twisted relative to the first [Palmer 2002]. Some examples of the horse-shoe-like effect is visually illustrated in Fig.1 with scRNA-seq datasets from the early stages of human embryonic development [Petropoulos et al. 2016], mouse preimplantation embryos [Deng et al. 2014] and liver haematopoiesis [Watson et al. 2022]. Similarly, the horse-shoe-like phenomenon can often be observed in PC plots of other scRNA-seq datasets [Cheng et al. 2019, Wagner et al. 2018, Yuan et al. 2019, Haghverdi et al. 2018].

The horse-shoe effect has been explored in various fields [Morton et al. 2017, Diaconis et al. 2008, Podani and Miklós 2002, De'ath 1999] to gain deeper insights about the dataset but remains largely unexplored in single cell studies. The limited literature on the horse-shoe-like effect in single-cell research interprets it as an artifact of dimensionality reduction [Hsu and Culhane 2020, Hsu and Culhane 2023]. In other fields, efforts have been made to offer logical explanations for the horse-shoe effect, aiming to showcase that this effect is more than just an artifact [Morton et al. 2017, Diaconis et al. 2008]. It is important to show that the interpretation of the horse-shoe-like effect as an artifact of dimensionality is not true for scRNA-seq data.

In this research work, we first used simulated scRNA-seq datasets to recapitulate the horse-shoe-like phenomenon and provide an intuitive explanation of its occurrence in PCA plots. We presented a systematic analysis of the horse-shoe-like effect from three different but inter-related horse-shoe perspectives. These perspectives include 1) Ordered band table gives rise to the horse-shoe effect [Diaconis et al. 2008], 2) Horse-shoe effect indicates the presence of a dominant gradient [Podani and Miklós 2002, De'ath 1999], and 3) Horse-shoe effect arises as a consequence of distance saturation property [Morton et al. 2017]. Next, we generated simulated dataset lacking horse-shoe-like effect to signify the absence of horse-shoe-like effect. Afterwards, we corroborated our findings with three real scRNA-seq datasets. We identify a potential problem with horse-shoe perspective III, an acknowledged explanation for the occurrence of the horse-shoe effect in PCA plots. Additionally, we also analyzed a mathematical model for the horse-shoe-like effect in the context of PCA and scRNA-seq data. Then, we compared the estimated solutions for eigenvectors with principal components of simulated and real datasets. Moreover, we showed that the horse-shoe-like effect in single-cell studies, often considered a result of dimensionality reduction, is not accurate. Finally, we briefly discussed a biological explanation for the outcomes of various horse-shoe perspectives in the context of the real scRNA-seq datasets..

Methods

Generating Simulated Datasets

Ordered band table is described as a data matrix that exhibits a distinct pattern where dense, non-zero values cluster along the diagonal, leaving the rest of the table sparsely populated [Morton et al. 2017]. In this regard, we first reproduced two simulated datasets that aligns with the description of the ordered band table. We call these simulated datasets as 'Simulated Band Dataset A' and 'Simulated Band Dataset B' for later reference. 'Simulated Band Dataset B' represents a

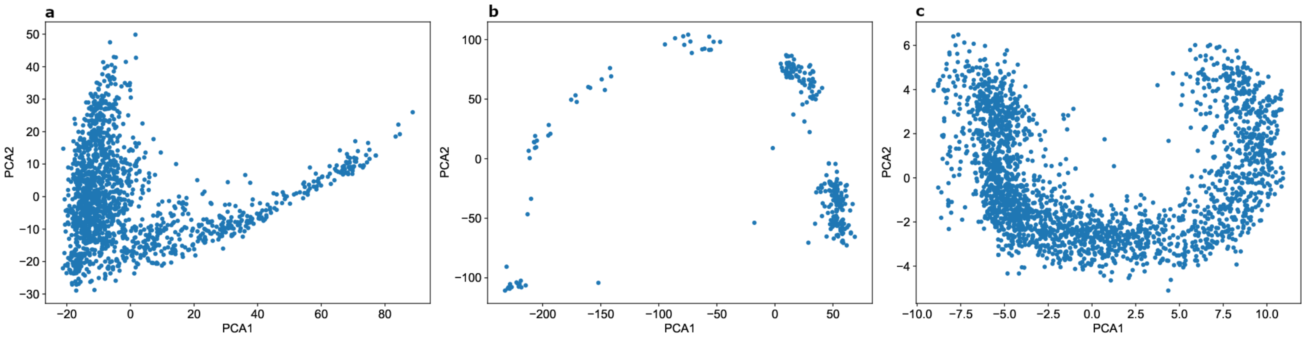

Fig. 1. (a) 2D PC plot of early human embryonic scRNA-seq dataset, (b) 2D PC plot of mouse embryonic preimplantation scRNA-seq dataset and (c) 2D PC plot of liver haematopoiesis scRNA-seq dataset with horse-shoe-like effect.

relatively more realistic band matrix. Next, we generated a simulated dataset with ordered blocks along diagonal instead of ordered bands. This simulated dataset with ordered blocks along diagonal represents a cell-gene matrix often observed in single-cell studies where cells of different types are sorted by marker genes [Zhang et al. 2014].

Applying PCA to observe horse-shoe-like effect

We applied PCA on datasets to observe horse-shoe-like effect, thus verifying horse-shoe perspective I. We used the first two principal components, acquired from applying PCA on simulated datasets, to draw 2D PC plots.

Local and global principal curve algorithms to recover dominant gradient

In literature, the appearance of a horse-shoe effect is often linked to the presence of at least one dominant gradient in the dataset [Morton et al. 2017, Podani and Miklós 2002]. Dominant gradient is described as a spatially/temporally varying aspect of the environment/genes which is expected to be related to species/cell composition [Palmer 2002].

The method used in the field of ecology and microbial analysis to recover the underlying dominant gradient is by identifying a 'Principal Curve'. Principal curves are smooth lines that have a special property: every point on the curve is the average of all the data points that align with it [Hastie and Stuetzle 1989]. Principal curves are described as an ideally suited method for direct and indirect gradient analysis [De'ath 1999].In this study, we employed the local algorithm "Greedy Constraint Local Principal Curve" (CLPCG) [Chen et al. 2016] and global algorithm "Non Linear PCA" (NLPCA) [Kramer 1991] for principal curves to recover the dominant gradient. The recovered dominant gradient is observed to verify horse-shoe perspective II on both simulated and real datasets.

Constructing 2D plot to observe distance saturation property

In PCA, the euclidean distance measures 'local' similarity or proximity between points in the reduced-dimensional space, providing a quantitative measure of relationships between cell samples. We emphasize on the word 'local' because euclidean distances tend to become saturated. To demonstrate the saturation property of euclidean distances, we use 2D distance saturation plot.

To construct 2D distance saturation plot, we start by selecting the first column of a sorted or ordered band table, labelled as '0' [Morton et al. 2017]. Then euclidean distance is calculated between reference sample and all the samples within the dataset including reference sample itself. After this, column index array of samples in the ordered band table is used as x-axis entries and the corresponding distances are used as y-axis entries. Plotting scatter plot for these x-axis and y-axis entries results in 2D distance saturation plot. We used this plot to verify horse-shoe perspective III on both simulated and real datasets.

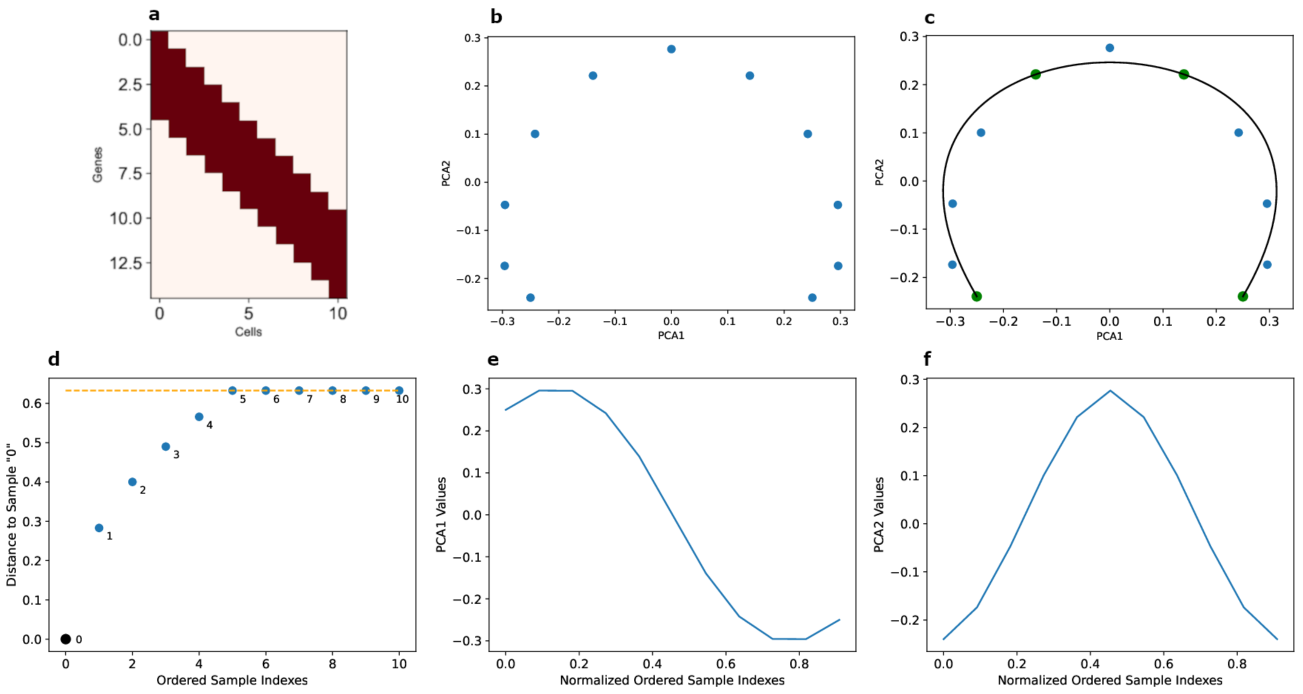

Fig. 2. (a) Ordered cell-gene data matrix, (b) 2D PC plot with horse-shoe-like effect, (c) Recovered dominant gradient superimposed on 2D PC plot, (d) 2D Distance saturation plot, (e) First eigenvector and (f) Second eigenvector for simulated band dataset A

Sorting gene expression matrix of real datasets to reveal ordered band table

The three scRNA-seq datasets used in this study, have a prominent horse-shoe-like effect in 2D PC plot as shown in Fig.1. To verify horse-shoe perspective I on these real biological datasets, the gene expression matrix is sorted and then observed for ordered band table along diagonal. Researchers in the field of ecology and microbial analysis use a method called 'niche_sort' to reveal ordered band table for real biological data [Morton et al. 2017]. This method requires two inputs: the gene expression data matrix and the dominant gradient. We used the information of embryonic time-points for human and mouse embryonic datasets and the cell stages of haematopoiesis for liver haematopoiesis dataset, as dominant gradient.

The mean values of the gradient for these scRNA-seq datasets are calculated using:

$$\bar{\phi_g} = \sum_{i=1}^{N} \phi_i \frac{g_i}{\sum_{j=1}^{M} g_j} \qquad (1)$$

In Eq. 1 $\bar{\phi_g}$ is the mean gradient of gene g. ϕ_i is the sample gradient of cell i. g_i is the counts or expression of gene g in sample i.

Mathematical Model for horse-shoe-like effect in PCA plots

Here, we summarized a mathematical model to explain horse-shoe-like effect in 2D PC plots. In the context of PCA, we describe estimated solutions for the first two principal components or eigenvectors. Then use the estimated principal components to plot 2D PC plot and observe horse-shoe-like effect.

In the context of single cell data, the fundamental assumption is that genes are evenly distributed. The implication of this assumption is that it allows for the identification or distinction of distances between cells in the dataset. Having a uniform distribution of genes makes it easier to measure and compare the distances between different cells, aiding in the analysis of the data. This leads to the following equation:

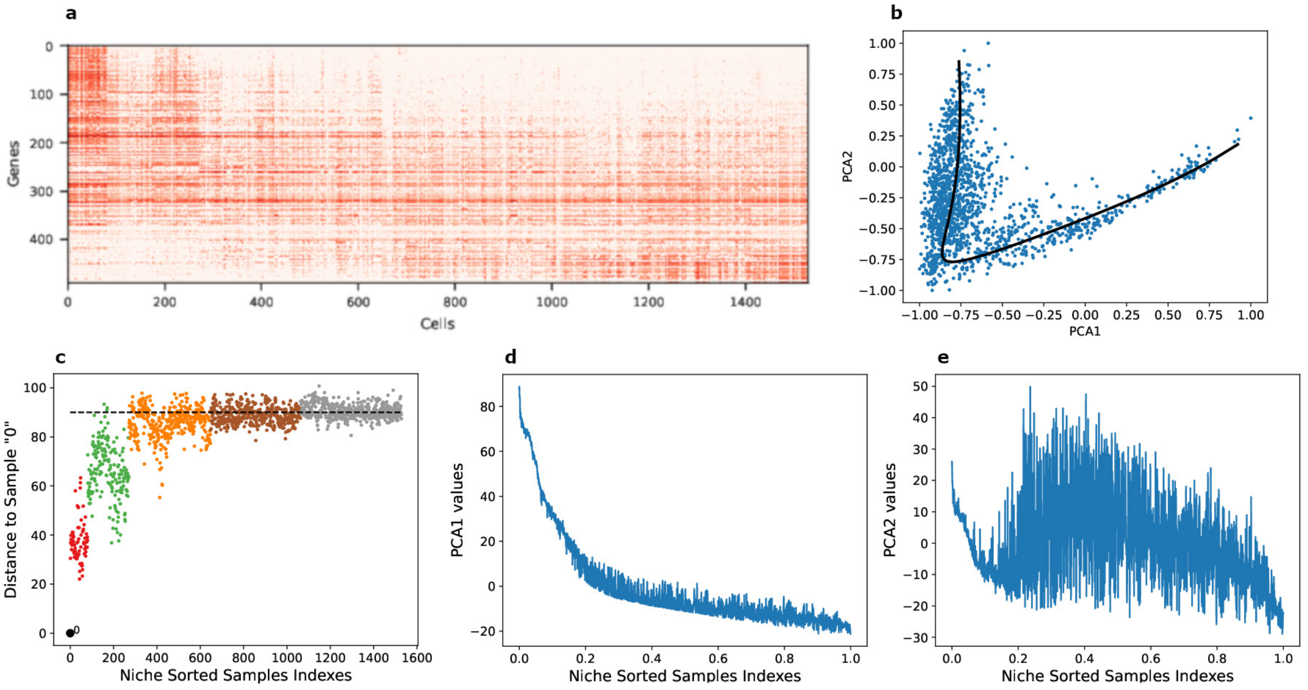

Fig. 3. (a) Sorted gene expression matrix, (b) Mapping of the estimated response curve on the corresponding 2D PC plot, (c) 2D distance saturation plot, (d) Principal Component 1 values and (e) Principal Component 2 values for early human embryonic dataset

$$d(x_i, x_j) = |\frac{i}{n} - \frac{j}{n}|. \tag{2}$$

In the above equation, x_i represents the position of cell i in gene expression matrix with uniform spacing ($x_i = i/n$). In a similar case, the authors of [Diaconis et al. 2008] use a measure of dissimilarity in the context of multi-dimensional scaling. In our case, the dissimilarity can be represented as:

$$X(i, j) = 1 - e^{(-d(x_i, x_j))}. \tag{3}$$

The resulting matrix that shows dissimilarity between samples is:

$$X = \begin{bmatrix} 0 & \cdots & 1 - e^{-(n-1)/n} \\ 1 - e^{-1/n} & \cdots & \vdots \\ \vdots & \ddots & 1 - e^{-1/n} \\ 1 - e^{-(n-1)/n} & \cdots & 0 \end{bmatrix} \tag{4}$$

Eq.4 represents the distance dissimilarity matrix, which is why the values along the diagonal are zeros and the exponential term representing similarity, is subtracted from 1. To provide a basic intuition for the formation of the distance dissimilarity matrix, we calculated the distance dissimilarity matrix for "Simulated Band Dataset A" and reported it in Table S1. Upon closer inspection, a similar pattern can be observed between Eq.4 and Table S1 with diagonal values as zero and increasing distance values away from diagonals.

Additionally, to gain basic intuition for the use of exponential terms in Eq.4, we plot column 1 from Table S1 alongside column 1 from Eq.4. These are demonstrated as subplots in Fig.S3.

In the context of PCA, we are interested in finding or estimating the eigenvectors and eigenvals for the above distance dissimilarity matrix. Next, by following established procedures, a kernel function is derived from dissimilarity matrix. This kernel function is subsequently employed to deduce functions for the first two eigenvectors or principal components [Diaconis et al. 2008, Santin and Schaback 2016]. The findings reported in [Diaconis et al. 2008] propose a trigonometric solution for these functions.

Subsequently, the approximated functions for the first and second eigenvectors can be expressed as Eq.5 and Eq.6,

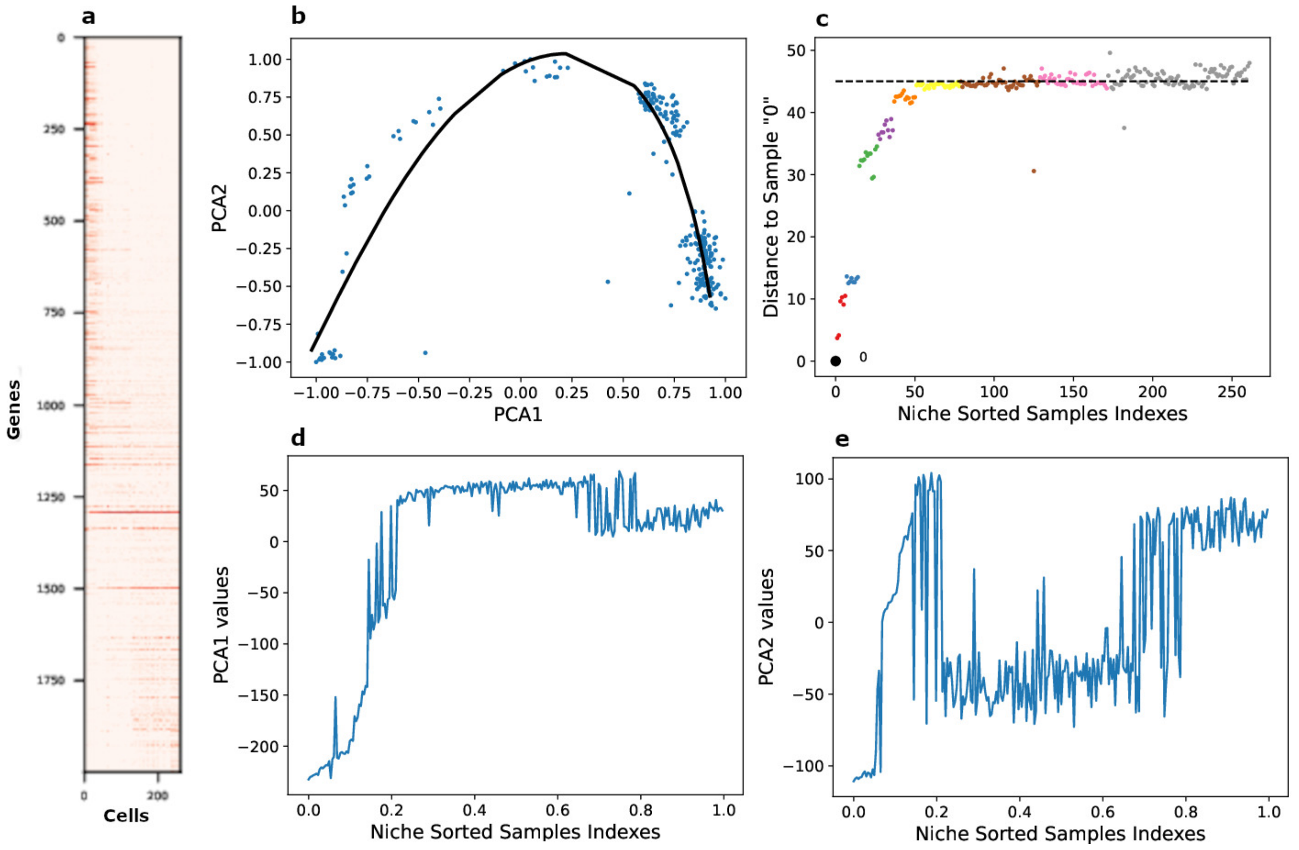

Fig. 4. (a) Sorted gene expression matrix, (b) Mapping of the estimated response curve on the corresponding 2D PC plot, (c) 2D distance saturation plot, (d) Principal Component 1 values and (e) Principal Component 2 values for mouse embryonic data

corresponding to eigenvalues of $\lambda_1 = 0.09$ and $\lambda_2 = 0.04$ respectively:

$$f_1(x_i) = f_{n,a_1}(x_i) = sin(3.1796(\frac{i}{n} - \frac{1}{2})) \quad (5)$$

$$f_2(x_i) = f_{n,a_2}(x_i) = cos(4.8989(\frac{i}{n} - \frac{1}{2})) \quad (6)$$

Finally, we construct a 2D PCA mapping using the first and second eigenvectors as follows:

$$f(x_i) = (\sqrt{\lambda_1} f_1(x_i), \sqrt{\lambda_2} f_2(x_i)) \quad (7)$$

Results

Simulated dataset with horse-shoe-like effect validates the three horse-shoe perspectives

The 2D PC plots for "Simulated Band Dataset A" and "Simulated Band Dataset B" depict a prominent horse-shoe-like effect as shown in Fig.2b and Fig.S2b, respectively. These results validate horse-shoe Perspective I on these simulated datasets.

Next, we explored horse-shoe Perspective II, on simulated band datasets A and B. In this regard, we used the local algorithm (CLPCG) of principal curves to recover the dominant gradient from these simulated datasets. Fig.2c and Fig.S2c show the recovered principal curve superimposed onto the 2D PC plot for "Simulated Band Dataset A" and "Simulated Band Dataset B," respectively. It can be observed that the recovered curves are a good representation of their corresponding simulated datasets.

Finally, we explore horse-shoe Perspective III on simulated datasets. To this end, we demonstrated the distance saturation property for ”Simulated Band Dataset A” in Fig.2d and ”Simulated Band Dataset B” in Fig.S2d. The distance saturation for both simulated datasets occur around the orange dotted line.

Simulated dataset lacking horse-shoe-like effect signifies its absence

Applying PCA on simulated dataset with ordered blocks (Fig.S1a) along primary diagonal instead of ordered band, lacks horse-shoe-like effect in the corresponding 2D PC plot (Fig.S1b). This simulated dataset gives a contrasting view and helps in better understanding the underlying mechanism that gives rise to horse-shoe-like effect. This dataset also validates that the presence of horse-shoe-like shape is a specific and meaningful occurrence in single-cell analysis rather than some artifact or random result.

Real scRNA-seq datasets corroborate our findings for the three horse-shoe perspectives.

Next, we generalized the acquired understanding of the three horse-shoe perspectives with the help of three real scRNA-seq datasets. These datasets include early human embryonic, mouse preimplantation embryos and liver haematopoiesis. First, we validated the three horse-shoe perspectives on early human embryonic scRNA-seq dataset. In this regard, we illustrated that the original unsorted data matrix can be sorted to reveal a band along the diagonal as shown in Fig.3a. This sorted gene expression data matrix gives rise to the horse-shoe-like effect in the real human embryonic dataset, thus validating horse-shoe perspective I. Next, we explored the horse-shoe perspective II on human embryonic dataset. In this regard, we first used the local CLPCG algorithm to recover the dominant gradient . However, upon inspection, the recovered dominant gradient appears to be a poor representation of the underlying dataset as shown in Fig.S4. To this end, we used the global algorithm NLPCA.

The implementation of the global algorithm NLPCA within the prinPy Python package is noted to be particularly well-suited for sparsely represented data compared to the local algorithm. Fig.3b shows that the recovered principal curve using NLPCA is a good estimation for the human embryonic dataset.

Afterwards, we construct 2D distance saturation plot for human embryonic scRNA-seq dataset in Fig.3c, to verify horse-shoe perspective III. It is evident that around black dotted line the distance saturation occurs for reference sample ‘0’.

Fig.4 and Fig.5 corroborates our findings for the three horse-shoe perspectives on mouse preimplantation embryonic and liver haematopoiesis datasets respectively. The main findings of our experimental results are summarized in Table 1.

Experimental Results Identify a Problem with horse-shoe Perspective III

The authors proposing that horse-shoe effect arises as a consequence of distance saturation property (perspective III), have explained the concept elegantly [Morton et al. 2017]. Supplementary section 2 provides brief explanation of the authors for distance saturation property in the context of horse-shoe effect. Here, we reproduced a subset of their simulation dataset to demonstrate that the distance saturation property lacks sufficient clarity. Fig.6 shows that despite the absence of distance saturation property, the horse-shoe-like effect still appears in the corresponding 2D PC plot. Furthermore, we supported our observation with additional experiment in Fig.S5 by increasing the number of samples to 20.

Based on our experimental results, we concluded that the horse-shoe-like effect can occur even when the distance saturation property is not present in the dataset. While the theory holds for the simulated and the real scRNA-seq datasets, it is important to note that there are cases where the theory does not hold. The two simulated datasets in Fig.6 and Fig.S5 are examples of such cases.

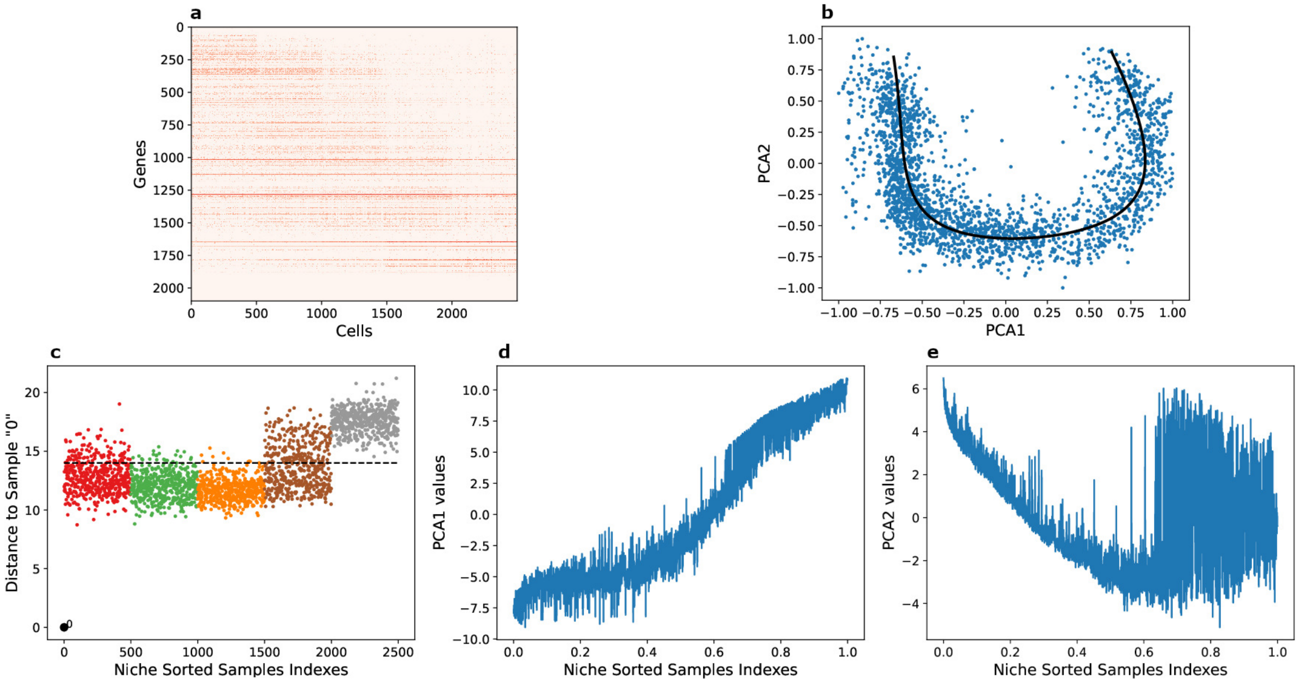

Fig. 5. (a) Sorted gene expression matrix, (b) Mapping of the estimated response curve on the corresponding 2D PC plot, (c) 2D distance saturation plot, (d) Principal Component 1 values and (e) Principal Component 2 values for liver haematopoiesis scRNA-seq dataset

Principal Components of Simulated and scRNA-seq Data Validate the Results of Mathematical Model

To visualize the results of the mathematical model summarized in section II, we first plotted the two estimated eigenvectors from Eq.5 and Eq.6 in Fig.S6a and Fig.S6b, respectively. These figures suggest trigonometric solutions to first and second eigenvectors. Following that, we created Fig.S6c to demonstrate the appearance of a horse-shoe-like pattern by plotting Eq.7.

Next, we plot the first two eigenvectors or principal components for 'Simulated Band Dataset A' in Fig.2e and Fig.2f and 'Simulated Band Dataset B' in Fig.S2e and Fig.S2f, respectively. Similar to the eigenvectors from mathematical model, the first two principal components from both simulated datasets are trigonometric. However, it can be observed that compared to 'Simulated Band Dataset A', the first two principal components for 'Simulated Band Dataset B' are relatively noisier. This observation can be attributed to fact that 'Simulated Band Dataset B' is relatively more complex.

Then, we visualized the first two principal components for the real human embryonic scRNA-seq dataset, ordered with the niche_sort method. The corresponding results are depicted in Fig.3d and Fig.3e respectively. The first two PCs represent a noisy trigonometric pattern, which is significantly more complex compared to the principal components of both simulated datasets. Similarly, Fig.4d, Fig.4e and Fig.5d, Fig.5e illustrates the noisy trigonometric pattern of first two principal components for mouse embryonic and liver hematopoiesis datasets respectively.

We also verified that the first two PCs within the two simulated datasets, used to explain the problem with the statement of horse-shoe perspective III, have trigonometric solutions as shown in Fig.S7.

Horse-shoe-like Effect on scRNA-seq Data is Not Merely an Artifact

The prominent horse-shoe-like effect observed in simulated and real datasets indicates a trigonometric relation of the second principal component to the first principal component, instead of a quadratic relation. This observation contradicts the notion that the horse-shoe-like effect is merely an artifact of reducing dimensionality. The trigonometric relationship between eigenvectors has been previously examined in

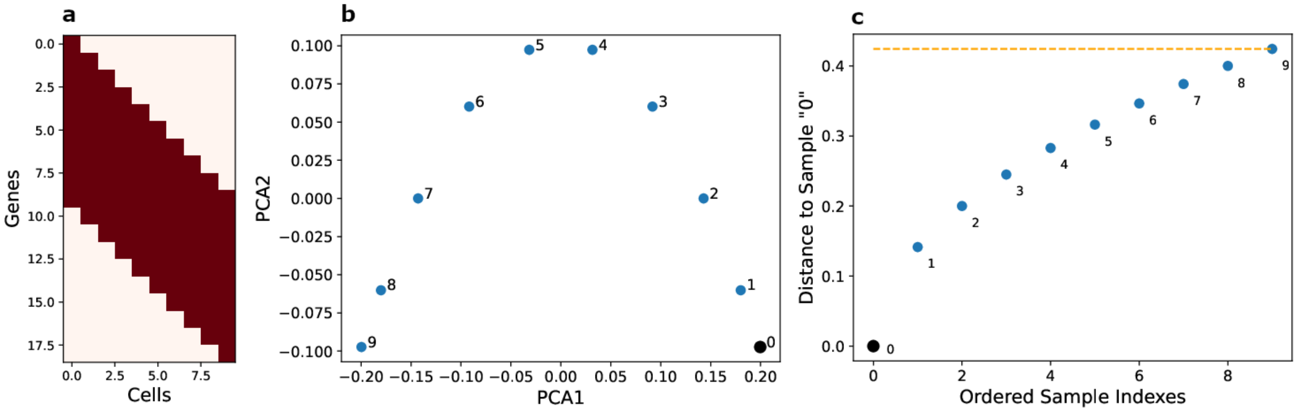

Fig. 6. (a) Simulated Band Data Matrix with samples that does not hold distance saturation property for any sample, (b) Corresponding 2D PC plot and (c) The distance saturation plot

[Diaconis et al. 2008] to counter the narrative of the horse-shoe effect as an artifact of dimensionality reduction.

Conclusion and Discussion

Our study contributes to the field of single-cell analysis by providing logical explanation for horse-shoe-like phenomenon. Initially, we showcased, using actual scRNA-seq datasets, the prevalence of the horseshoe-like effect as a recurrent phenomenon in single-cell analysis. Subsequently, we recapitulated this phenomenon using simulated datasets to offer a comprehensive understanding of its occurrence. Our approach illuminates the horse-shoe-like phenomenon with the help of three different but inter-related horse-shoe perspectives. After this, we generated simulated dataset lacking hose-shoe-like phenomenon to signify its absence. Finally, we corroborated the findings with real biological scRNA-seq datasets. We identified a potential problem or lack of clarity for a well-known theory attributed to induce horse-shoe phenomenon. Furthermore, we analyzed mathematical model and relate the model to simulated and real datasets in the context of horse-shoe-like phenomenon. We also discussed that the horse-shoe-like effect is not an artifact of dimensionality.

In a biological context, the ordered band tables in Fig.3a, 4a, and 5a illustrates the shift in gene expression patterns across developmental stages: from E3 to E7 in early human embryonic development, from MIIoocyte to late blast in mouse embryonic development, and from haematopoiesis stem cells and multipotent progenitors (HSC/MPPs) to late erythroid cells in liver haematopoiesis. These ordered band tables effectively captured the progression or growth of cellular maturation from an early state to a more advanced stage. These observations are further substantiated by the recovered dominant gradient presented in Fig.3b, Fig.4b and Fig.5b for human embryonic, mouse embryonic and liver haematopoiesis datasets respectively. Moreover, an examination of Fig.3c, Fig.4c and Fig.5c reveals a distinct distance saturation pattern for reference sample '0' of the above mentioned scRNA-seq datasets. The reference sample belongs to E3 in human embryonic dataset, MIIoocyte in mouse embryonic dataset and HSC/MPP in liver haematopoiesis dataset from their respective niche sorted ordered band tables. Notably, the distance saturation phenomenon is evident for sample '0' around the black dotted line. A closer examination of Fig.3c for human embryonic dataset reveals that the samples along the line, where the distance from the reference sample is maximized, correspond to either E5, E6, or E7. To be more precise, these samples consist of a subset of E5 samples and all samples from E6 and E7. The distance saturation for sample '0' occurs primarily due to the scarcity of common genes/features. This pattern can be attributed to the fact that all samples in E3 belong to pre-lineage cell type, while only a subset of E5 samples are affiliated with pre-lineage cell types. Additionally, pre-lineage cell types are absent in both

E6 and E7 [Petropoulos et al. 2016]. Similar trends in distance saturation can be identified in the mouse embryonic and liver haematopoiesis datasets.

In essence, the key findings of our research are that scRNA-seq datasets with gradual or continuous differentiation in the cell states depict horse-shoe-like effect in the corresponding 2D PC plot. We further emphasized that the horse-shoe-like effect is an interpretable feature of PCA analysis and not a random result. Upon observing horse-shoe-like effect in a scRNA-seq dataset, the logical implications are 1) there is an ordered band of expressed genes along primary diagonal when the data matrix is sorted properly. 2) the dataset is influenced by a dominant gradient in the form of sequencing timepoints or gradual state transitions of single cells, 3) The dataset may potentially be subjected to distance saturation property. However, it is still possible that the underlying dataset lacks distance saturation property.

The presence of the horse-shoe-like effect suggests a developmental trajectory characterized by continuous transitions between cell states, as opposed to discrete clusters. Recognizing this effect can direct subsequent analyses, encouraging the use of pseudo-time analysis method to further explore the biological processes at play. Notably, the pseudo-time analysis method Slingshot [Street et al. 2018] employs principal curves, as utilized in this study for recovering the dominant gradient, to draw the paths for each cell lineage.

We conducted additional experiments to understand how PCA saturation relates to the rate of zero entries and the rank of the matrix. In Fig.S9, we observed that PCA saturation happens slightly faster for liver haematopoiesis, human embryonic, and mouse embryonic datasets, in the order mentioned. Interestingly, these trends match the rates of zero entries in the datasets. Liver haematopoiesis, with the highest rate of zero entries at 91.57%, achieves saturation most quickly, followed by the human embryonic dataset at 59.81%, and the mouse embryonic dataset at 53.52%. Additionally, the rank of the dataset matrices follows a similar pattern, with liver haematopoiesis having the highest rank of 1937, followed by the human embryonic dataset at 490, and the mouse embryonic dataset at 262. This correlation between PCA saturation, zero entry rates, and matrix rank suggests that data sparsity may influence dimensionality reduction.

Ethics approval and consent to participate

No ethics approval was required for the study.

Availability of data and materials

The original scRNA-seq data of early human embryonic cells can be found at E-MTAB-3929.

The mouse embryonic dataset is publicly available from the Gene Expression Omnibus (GEO) with the accession number GSE45719.

The Liver Haematopoiesis dataset is publicly available from Developmental Human Cell Atlas

The code for reproducing the results of this study is available at: https://github.com/najeebullahshah/PCA-Horse-Shoe

Consent for publication

Not Applicable

Competing interests

The author declares no competing interests.

Funding

This work is partially supported by NSFC grant 62250005. The authors extend special gratitude to CSC (Chinese Scholarship Counsil) for their invaluable support, without which this research would not have been possible.

Authors' contributions

XZ conceived the project. NS designed and conducted the experiments and summarized the mathematical model. QM helped in analyzing the experimental results. ZZ helped in

Table 1. Summary of Horse-shoe-like Perspectives on Two Simulated Datasets and Three Real scRNA-seq Datasets

Dataset Type	Dataset Name	Horse-shoe Perspective	Main Finding
Simulated Datasets	Simulated Band Dataset A	Perspective I	2D PC plot shows horse-shoe effect validates perspective I (Fig.2b)
		Perspective II	CLPCG recovers dominant gradient validating perspective II (Fig.2c)
		Perspective III	2D distance saturation plot validates perspective III (Fig.2d)
	Simulated Band Dataset B	Perspective I	2D PC plot shows horse-shoe effect validates perspective I (Fig.S2b)
		Perspective II	CLPCG recovers dominant gradient validating perspective II (Fig.S2c)
		Perspective III	2D distance saturation plot validates perspective III (Fig.S2d)
Real scRNA-seq datasets	Early Human Embryonic scRNA-seq dataset	Perspective I	Neiche sorting reveals ordered band table validating perspective I (Fig.3a)
		Perspective II	NLPCA recovers dominant gradient validating perspective II (Fig.3c)
		Perspective III	2D distance saturation plot validates perspective III (Fig.3d)
	Mouse Preimplantation Embryos	Perspective I	Neiche sorting reveals ordered band table validating perspective I (Fig.4a)
		Perspective II	NLPCA recovers dominant gradient validating perspective II (Fig.4c)
		Perspective III	2D distance saturation plot validates perspective III (Fig.4d)
	Liver Haematopoiesis	Perspective I	Neiche sorting reveals ordered band table validating perspective I (Fig.5a)
		Perspective II	NLPCA recovers dominant gradient validating perspective II (Fig.5c)
		Perspective III	2D distance saturation plot validates perspective III (Fig.5d)

the mathematical model. NS wrote the manuscript, which was subsequently reviewed and verified by all authors.

Acknowledgments

We thank the anonymous reviewers for their suggestions that have helped to improve this work.

References

Chen, D. *et al.* (2016). Constraint local principal curve: Concept, algorithms and applications. *Journal of Computational and Applied Mathematics*, 298, 222–235.

Cheng, S. *et al.* (2019). Single-cell RNA-seq reveals cellular heterogeneity of pluripotency transition and X chromosome dynamics during early mouse development. *Cell Reports*, 26(10), 2593–2607.

Deng, Q., *et al.* (2014). Single-cell RNA-seq reveals dynamic, random monoallelic gene expression in mammalian cells. *Science*, 343(6167), 193–196.

De'ath, G. (1999). Principal curves: a new technique for indirect and direct gradient analysis. *Ecology*, 80(7), 2237–2253.

Diaconis, P., *et al.* (2008). Horseshoes in multidimensional scaling and local kernel methods. *The Annals of Applied Statistics*.

Haghverdi, L., *et al.* (2018). Batch effects in single-cell RNA-sequencing data are corrected by matching mutual nearest neighbors. *Nature Biotechnology*, 36(5), 421–427.

Hastie, T., and Stuetzle, W. (1989). Principal curves. *Journal of the American Statistical Association*, 84(406), 502–516.

Hsu, L. L., and Culhane, A. C. (2020). Impact of data preprocessing on integrative matrix factorization of single cell data. *Frontiers in Oncology*, 10, 973.

Hsu, L. L., and Culhane, A. C. (2023). Correspondence analysis for dimension reduction, batch integration, and visualization of single-cell RNA-seq data. *Scientific Reports*, 13(1), 1197.

Huang, H., *et al.* (2022). Towards a comprehensive evaluation of dimension reduction methods for transcriptomic data visualization. *Communications Biology*, 5(1), 719.

Kramer, M. A. (1991). Nonlinear principal component analysis using autoassociative neural networks. *AIChE Journal*, 37(2), 233–243.

McInnes, L., *et al.* (2018). UMAP: Uniform manifold approximation and projection for dimension reduction. *arXiv preprint arXiv:1802.03426*.

Morton, J. T., *et al.* (2017). Uncovering the horseshoe effect in microbial analyses. *Msystems*, 2(1), 10–1128.

Palmer, M. (2002). A glossary of ordination-related terms. URL https://ordination.okstate.edu/glossary.htm.

Petropoulos, S., *et al.* (2016). Single-cell RNA-seq reveals lineage and X chromosome dynamics in human preimplantation embryos. *Cell*, 165(4), 1012–1026.

Podani, J., and Miklós, I. (2002). Resemblance coefficients and the horseshoe effect in principal coordinates analysis. *Ecology*, 83(12), 3331–3343.

Santin, G., and Schaback, R. (2016). Approximation of eigenfunctions in kernel-based spaces. *Advances in Computational Mathematics*, 42(4), 973–993.

Street, K., *et al.* (2018). Slingshot: cell lineage and pseudotime inference for single-cell transcriptomics. *BMC Genomics*, 19, 1–16.

Van der Maaten, L., and Hinton, G. (2008). Visualizing data using t-SNE. *Journal of Machine Learning Research*, 9(11).

Wagner, D. E., *et al.* (2018). Single-cell mapping of gene expression landscapes and lineage in the zebrafish embryo. *Science*, 360(6392), 981–987.

Watson, E. R., *et al.* (2022). How does data structure impact cell-cell similarity? Evaluating the influence of structural properties on proximity metric performance in single cell RNA-seq data. *bioRxiv*, 2022–04.

Yuan, W., *et al.* (2019). S100A4 upregulation in PIK3CA H1047R; TRP53 R270H; MMTVcre-driven mammary tumors promotes metastasis. *Breast Cancer Research*, 21, 1–11.

Zhang, Y., *et al.* (2014). An RNA-sequencing transcriptome and splicing database of glia, neurons, and vascular cells of the cerebral cortex. *Journal of Neuroscience*, 34(36), 11929–11947.
